# Primary umbilical hernia repair: does suture type matter?

**DOI:** 10.1007/s00464-025-12212-3

**Published:** 2025-09-17

**Authors:** Alex Finn, Kristin Hawes, Tien Hua, Madi Mangione, Elizabeth Martin, Stephen Kuselias, Giuseppe Zambito, Amy Banks-Venegoni

**Affiliations:** 1 Corewell Health General Surgery Residency, Michigan State University, 100 Michigan St NE, MC 188, Grand Rapids, MI 49505 USA; 2 Michigan State University College of Human Medicine, Grand Rapids, USA; 3 Corewell Health Department of General Surgery, Grand Rapids, USA; 4 Providence College Rhode Island, Providence, USA

**Keywords:** Umbilical, Hernia, Primary repair

## Abstract

**Background:**

Umbilical hernias are a common surgical pathology. Umbilical hernias 2 cm or less are generally treated with open primary suture repair with associated risk of recurrence up to 27 percent. A variety of suture and repair techniques are used with little evidence to suggest which provides the lowest risk of recurrence. The primary goal of our study is to evaluate recurrence rates between primary umbilical hernia repairs with braided and monofilament suture.

**Methods:**

We conducted a retrospective review of primary umbilical hernia repairs in patients over the age of 18 performed at a single institution by a group of ten surgeons from November 2018 to November 2023. The primary outcome was comparison of recurrence rates between braided and monofilament suture. Secondary outcomes include recurrence rate of monofilament absorbable versus permanent repair, recurrence rate by repair method, rates of wound infection and suture granulomas.

**Results:**

We evaluated 797 patients that met inclusion criteria with a mean age of 50.9 years, mean BMI 29.6, mean CCI 1.64, and 77% male. We had a 46-month average follow-up via chart review. Mean hernia size was 1.11 cm. 86.4% were repaired with braided (Ethibond) and 13.6% with monofilament (PDS or Prolene) suture. Recurrence rate was 3.9% versus 5.6% for braided and monofilament, respectively, with no significant difference. The incidence of wound complications was not significant between the two groups. There was an increased recurrence rate using the pants over vest repair technique.

**Conclusion:**

Our study indicates that the type of suture used in primary umbilical hernia repairs does not significantly impact the rates of recurrence, wound infection, or suture granuloma formation. However, the pants-over-vest technique is associated with a higher recurrence rate.

Umbilical hernias are extremely common, estimated to be prevalent in 23–50 percent of people [1]. They account for approximately 6% to 14% of all abdominal wall hernias in adults. 90% of adult umbilical hernias are acquired due to increased intra-abdominal pressure from obesity, ascites, pregnancy, or chronic cough [2]. Surgical repair of umbilical hernias is recommended for worsening pain, increase in size, and/or bowel obstructive symptoms. Primary suture repair is the general recommendation for small umbilical hernias, specifically those two centimeters or less in size [3, 4]. However, the risk of hernia recurrence with primary umbilical repair is not insignificant, estimated to be anywhere from 4.9 to 27 percent [5, 6]. The rate of recurrence appears to be related to the dimensions of the hernia, with smaller defects tending to have lower recurrence rate following repair [2, 7, 8].

Currently, there is little literature to support what suture type will provide the best repair with lowest recurrence risk for primary umbilical hernias. There are a myriad of suture types used for repair. Most commonly used sutures include monofilament absorbable (polydioxanone suture (PDS)), monofilament permanent (Prolene), and braided (Ethibond) suture. Several studies have been conducted to determine which suture is best to use when closing the abdominal cavity with surgeries such as laparotomies [9, 10]. However, in the setting of small primary umbilical hernias little is known about optimal suture choice for repair. While many surgeons use non-absorbable suture, there are also those who use absorbable suture in these cases with reportedly good outcomes [7].

Our study’s primary outcome aims to identify if there is less recurrence based on suture type. Secondary outcomes were evaluated looking for recurrence rates between repair techniques as well as comparisons between permanent and absorbable monofilament suture. In addition, we evaluated the rate of suture granulomas and wound infections.

## Materials and methods

We conducted a retrospective review of primary umbilical hernia repairs in patients performed at a single institution by a group of ten surgeons over a six-year period. Patients were identified as having an umbilical hernia by current procedural terminology (CPT) code. Consecutive surgeries were collected from November 1st, 2017, to November 1st, 2023. Exclusion criteria included incorrect current procedural terminology (CPT) codes, use of mesh, re-operative field, patients less than 18 years of age, and emergency surgery. Institutional review board approval was granted for this study.

The primary outcome was comparison of recurrence rates between braided and monofilament suture. Secondary outcomes included recurrence rate of absorbable and permanent monofilament suture, recurrence rate by repair method, rates of wound infection within thirty days, and suture granuloma formation. Charts were reviewed for recurrence defined either radiographically or by re-operative repair. Granuloma and infection rates were also recorded. Our follow-up time period was an average of 46 months via chart review.

### Statistical analysis

We performed our statistical analysis with recurrence as our main dependent variable using a chi-square test. As a follow-up for each test, we performed a binomial logistic regression with the following control variables: sex, BMI, smoker status, ASA class, hypertension, CCI. We included these as a robustness test and to control for alternative explanations.

## Results

### Primary outcomes

A total of 986 cases were identified, with 797 meeting inclusion criteria.^1^ The mean patient age was 50.9, mean BMI 29.6, mean CCI 1.6, and 77% were male. The average hernia defect size was 1.1 cm.^2^ We first compared the monofilament and braided groups using a chi-square test and found a significant difference between groups in the categories of age and gender (see Table 1, Panel A for descriptives). The rate of recurrence was lower for braided (3.95%) compared to monofilament (5.60%), but there was no significant difference between the two (*p* = 0.434; Table 1, Panel B). Binary logistic regression with the included covariates to reduce the error variance and test the robustness of the non-significant finding above was performed and still showed no significance (b = 0.414, *p* = 0.380). Of our covariates, we found that BMI does have a marginally significant relationship on recurrence (coefficient = 0.54, *p* = 0.087, two-tailed). The other covariates did not have a significant effect on recurrence (all p’s > 0.10). As a robustness test, we also included the identity of the surgeon as dummy coded covariate. The inclusion of this variable did not qualitatively change the results discussed above.

**Table 1:** Primary investigation

Panel A: Descriptives			
Mean [s.d.]	Braided *n* = 689	Monofilament *n* = 108	Differences *p*-values
Gender (female)	21.04% [0.41]	29.63% [0.46]	0.061
Age	51.26 [14.60]	48.22 [13.82]	0.043
BMI	29.74% [5.34]	28.96% [5.58]	0.163
Current smoker	11.03% [0.32]	7.41% [0.26]	0.313
Charleston Comorbidity Index (CCI)	1.67 [1.98]	1.38 [1.87]	0.147
History of hypertension	40.0% [0.49]	31.0% [0.46]	0.057

Panel B: Braided vs Monofilament recurrence			
n (frequency) [% of column total]	Braided	Monofilament	Total
No recurrence	662 [96.1%]	102 [94.4%]	764 [95.9%]
Recurrence	27 [3.9%]	6 [5.6%]	33 [4.1%]
Total	689 [100%]	108 [100%]	797 [100%]

### Secondary outcomes

We ran a chi-square test of absorbable (PDS) versus permanent (Prolene) monofilament sutures (n = 108, PDS vs Prolene). PDS had a slightly higher recurrence rate (6.8%) compared to Prolene (4.1%), but no significant difference was found between the two (*p* = 0.687) (Table 2, Panel A). Follow-up binary logistic regression that included covariates did not find a significant relationship in recurrence rates between PDS and Prolene (b = 0.394, *p* = 0.667).

**Table 2:** Secondary investigations

Panel A: PDS vs prolene recurrence			
*n* (frequency) [% of column total]	PDS (absorbable)	Prolene (permanent)	Total
No recurrence	55 [93.2%]	47 [95.9%]	102 [94.4%]
Recurrence	4 [6.8%]	2 [4.1%]	6 [5.6%]
Total	59 [100%]	49 [100%]	108 [100%]

Panel B: Figure of eight vs. pants over vest vs simple interrupted recurrence				
*n* [% of column total]	Figure of eight	Pants over vest	Simple interrupted	Total
No recurrence	431 [96.4%]	65 [90.3%]	268 [96.4%]	764 [95.9%]
Recurrence	16 [3.6%]	7 [9.7%]	10 [3.6%]	33 [4.1%]
Total	447 [100%]	72 [100%]	278 [100%]	797 [100%]

Panel C: Frequency of wound infection and suture granulomas			
*n* (frequency) [% of column total]	Braided	PDS (absorbable)	Prolene (permanent)
Granuloma	1 [0.1%]	1 [1.7%]	0 [0%]
Wound infection	5 [0.9%]	2 [3.4%]	0 [0%]
Total	689* [100%]	59 [100%]	49 [100%]

^*^Braided dataset was missing data points for both wound infection rates (*n* = 2) and granuloma (*n* = 1). Therefore, while there are 689 patients, missing datapoints decrease this total slightly depending on the dependent variable.,

Among repair techniques, recurrence rates differed. Our data was filtered for three techniques: figure of eight, simple interrupted, and pants over vest. The rate of recurrence for pants over vest (9.7%) was directionally higher than both figure of eight and simple interrupted (both 3.6%) (Table 2, panel B). The statistical test for these differences was significant (*p* = 0.045). Therefore, we added the surgical repair type as a categorical variable (in addition to suture type) in a binary logistic regression with the included covariates. In this analysis we found a trend toward significance between pants over vest versus simple interrupted (b = − 0.97 = 8., *p* = 0.070) and a significant difference for pants over vest versus figure of eight (b = − 1.13, *p* = 0.025). Again, we find in this model a marginally significant effect of the covariate BMI on recurrence (p = 0.051) and no other significant covariates (all p’s > 0.30). We do note that, when we run a robustness check with the surgeon who performed the surgery added as a covariate, the repair type variable is no longer significant for either comparison. We believe it is likely that the addition of surgeon variable greatly increases the number of variables included in the binomial regression model (over 20 for a dataset where there are only 33 recurrences out of 797) and that is causing a loss of statistical power in our test.

Finally, we examined both the suture granuloma rate and wound infection rate between braided and monofilament suture type (Table 2, panel C). We found that there was no significant difference in suture granuloma rates (*p* = 0.253) or for wound infection rates (*p* = 0.244).

## Discussion

Current literature is lacking on direct evaluation of primary umbilical hernias repaired without mesh. There has been ample research evaluating which hernia defects would benefit from a mesh repair, as well as the best method of closing large abdominal incisions such as laparotomies [3, 4, 9, 10]. However, this does not answer what suture and repair technique is best for primary symptomatic umbilical hernias too small to be repaired with mesh. In this circumstance our findings suggest that suture type does not significantly impact recurrence rates. We also find that the inclusion of patient demographic factors does not impact this finding.

Importantly, we found the pants-over-vest repair technique demonstrated a higher recurrence rate compared to figure-of-eight technique. There was a trend toward a significant difference in increased recurrence for pants over vest compared to the simple interrupted repair as well. This may be due to specific surgeon technique or due to previously described undue tension from this repair type. These findings warrant further investigation.

## Limitations

This study is limited by its retrospective design and reliance on medical records, including CT scans and clinical follow-up for recurrence identification. Additionally, as a single-center study with low risk surgical candidates, generalizability is limited. A multi-institutional study with longer and more robust follow-up would be beneficial to validate these findings. Our data was also comparatively low in monofilament suture compared to braided (Ethibond). Many of our surgeons have made a transition toward monofilament absorbable repair in the last five years and collecting data going forward will increase the power for this population.

## Conclusion

Suture type does not significantly affect recurrence, suture granuloma formation, or infection rates in primary umbilical hernia repair. However, the pants-over-vest repair technique had a higher recurrence risk. Surgeons should feel confident using any suture type for small, uncomplicated primary umbilical hernia repairs but may consider avoiding pants-over-vest repairs. Future studies with larger sample sizes and multicenter data would enhance the generalizability of these findings.

## Footnotes

1 When we performed our statistical analysis, some patients were dropped because they were missing data on certain measures.

2 Based on 742 recorded cases from the initial sample size.
